# Prevention and treatment effect of total flavonoids in *Stellera chamaejasme L.* on nonalcoholic fatty liver in rats

**DOI:** 10.1186/s12944-015-0082-6

**Published:** 2015-08-06

**Authors:** Yu Wang, Jian-Yun Li, Min Han, Wen-Long Wang, Yun-Zhang Li

**Affiliations:** Ministry of Agriculture Key Laboratory of Clinical Diagnosis and Treatment Technology in Animal Disease, College of Veterinary Medicine, Inner Mongolia Agricultural University, 010018 Hohhot, Inner Mongolia China; Inner Mongolia Center for Endemic Disease Control and Research, 010031 Hohhot, Inner Mongolia China

**Keywords:** *Stellera chamaejasme L.*, Total flavonoids, Nonalcoholic Fatty Liver, Rats, Blood lipid, Blood lipid index, Liver index, Gene expression

## Abstract

**Background:**

The incidence of nonalcoholic fatty liver disease (NAFLD) has been increasing worldwide in parallel with the obesity epidemic. This study aims to investigate the effects of the total flavonoids in *Stellera chamaejasme L.* (TFSC) on the experimental NAFLD in high fat diet fed (HFD) rats.

**Methods:**

NAFLD model was induced in male Wistar rats by high-fat diet, and the rats in NAFLD group were randomized into NAFLD group (*n* = 20) and TFSC-treated group (*n* = 60). Both groups were given high-fat diet, and the normal group (*n* = 20) was given normal diet. In addition, the TFSC treated group was administered TFSC orally once a day at a low dose of 100 mg/kg (*n =* 20), medium dose of 200 mg/kg (*n* = 20), and high dose of 400 mg/kg (*n* = 20) for 6 weeks. Subsequently, the rats were sacrificed and body weight changes, lipid profiles in plasma and liver pathology were examined. The relative levels of fatty acid synthesis and β-oxidation gene expression in hepatic tissues were measured by quantitative real-time polymerase chain reaction (RT-PCR).

**Results:**

After the HFD administration for 4 weeks, the body weight,serum TC and TG levels in the rat of model group were significantly higher than in normal group (*P* < 0.05), and which Showed that the experimental NAFLD model was successfully established. While continual feeding with HFD deteriorated NAFLD and hyperlipidemia, and treatment with the different doses of TFSC effectively improved serum and liver lipid metabolism and liver function. A linear relationship between the dose of TFSC and blood lipid level was observed. The mRNA expression of hepatic acetyl-CoA carboxylase (ACC), fatty acid synthase (FAS), Leptin (LEP) and sterol regulatory element binding protein (SREBP)-1c as well as peroxisome proliferator-activated receptor (PPAR) –γ were significantly lower in high-dose group compared to the positive control group (*P* < 0.05). The hepatic mRNA expression of Cholesterol 7α-hydroxylase1 (CYP7A1), Carnitine palmitoyltransferase-1 (CPT1) and peroxisome proliferator-activated receptor (PPAR) –α were significantly higher in the high-dose group compared to the positive control group (*P* < 0.05). However, no difference was detected in the middle-dose group or the low-dose group compared to the positive control group (*P* > 0.05).

**Conclusion:**

TFSC treatment effectively improved NAFLD-related hyperlipidemia and inhibited liver steatosis in rats, and accompanied by modulating the expression of genes for regulating lipid metabolism.

## Introduction

Non-alcoholic fatty liver disease (NAFLD) is a common hepatic disease. Pathologically, it can present as simple steatosis, non-alcoholic steatohepatitis (NASH), and eventually progress to cirrhosis, an end-stage liver disease [1, 2]. The NAFLD is the most common cause of chronic liver disease in western world, and it has been increasing in China in the past years. The prevalence of NAFLD ranges from 6 % to 14 % in different populations [3, 4]. The median prevalence of ultrasonographic steatosis in Chinese populations is about 10 %, with a wide range of 1 % to more than 30 % [5]. Notably, 1 %–5 % of patients with simple steatosis can eventually develop actual cirrhosis, and 10 % to 15 % of patients with NASH can progress to cirrhosis and even to hepatocellular carcinoma [2].

In recent years, many synthetic drugs have been used to treat NAFLD problems. With various mechanisms of action, these drugs have different side effects, some of which could lead to severe complications. There has been an active effort of screening traditional Chinese medicine (TCM) to treat this fatal diseases [6].Our study aims to investigate the potential of *Stellera chamaejasme L.*, a type of TCM, in preventing and treating NAFLD.

*Stellera chamaejasme L.* is a plant in the family Thymelaeaceae. It grows widely in mountain slopes, grassland, and valleys in northern and western China [7]. *Stellera chamaejasme L.*, particularly the root, is toxic. Eating *Stellera chamaejasme L.* causes diarrhea, vomiting, and possible death in the livestock of these areas [8]. However, the roots of *Stellera chamaejasme L.*, called Langdu in traditional Chinese medicine, have long been used to expel water retention, clear phlegm, relieve masses, and destroy parasites [9]. Studies to identify and isolate individual compounds from *Stellera chamaejasme L.* have shown that the main chemical components of *Stellera chamaejasme L.* include flavonoids, coumarins, diterpenes, triterpenes, lignins, and phenylpropanoid glucosides [10, 11]. A recent study showed a low acute toxicity of total flavonoids extracted from *Stellera chamaejasme L.* with the median lethal dose (LD_50_) of 1.9848 g/kg in Balb/C mice, suggesting the total flavonoid extract is a promising natural medicine in drug development [12].

To the best of our knowledge, no studies has investigated the effects of dietary supplementation with *Stellera chamaejasme L.* stem extracts on fat metabolism in a high-fat diet (HFD)-induced NAFLD model. In this study, we examined the effects of TFSC on lipid levels and cholesterol levels in the liver and serum of rats fed with HFD, and on expression of genes involved in fatty acid synthesis and oxidation in the liver.

## Materials and methods

### Extraction and isolation

Total flavonoids were extracted from *Stellera chamaejasme L.* using ultrasonic extraction following the procedures detailed by Wu et al. [13]. Briefly, 300 g *Stellera chamaejasme L.* powder was soaked in 95 % EtOH, and then was extracted 3 times using ultrasound for 30 min each time at 50 °C with the solid–liquid ratio of 1:30. The suspension was concentrated under reduced pressure and then extracted with 50 mL petroleum ether (PE) 3 times. The PE extract was concentrated under reduced pressure to yield a dark concrete. Fifty mL distilled water was added to the concentrate, which was then heated in a water bath at 60 °C for 2 h, allowed to stand until it reached room temperature, and then filtered. The residue was taken and the processes of adding distilled water, heating in a water bath, and filtering were repeated 3 times.The residue was then evaporated.

### Measurement of total flavonoids

The total flavonoid content was determined as previously described [13] with slight modifications. Briefly, 0.25 mL of SBS extracts (100 μg/mL) was added to a tube containing 1 mL of double-distilled water. Next, 0.075 mL of 5 % NaNO_2_, 0.075 mL of 10 % AlCl_3_, and 0.5 mL of 1 M NaOH were added sequentially at 0, 5, and 6 min. Finally, the volume of the reacting solution was adjusted to 2.5 mLwith double-distilled water. The solution had an absorbance of 510 nm, which was detected using an Ultrospec TU-1901 Pro Spectrophotometer (Purkinje General, china). The final concentrate was the total flavonoid extract of 27.5 % content.

### Animals

Normal Male Wistar rats weighing 160–180 g were purchased from Inner Mongolia University Experimental Animal Center. Ten rats were placed in one cage and maintained under controlled room temperature (20 ± 2 °C) and relative humidity (50–70 %) with day/night cycle (12 h/12 h). All rats had free access to food and water. The animal care and study protocols were maintained in accordance with the Provisions and General Recommendation of Chinese Experimental Animals Administration Legislation [3].

### Treatment of animals

Rats were fed with normal pellet diet (NPD) and water ad libitum for 7 days to stabilize their metabolic condition. After the 1-week adaptation, the rats were randomly divided into NPD group (*n* =40) and NAFLD group (*n* = 100) randomly. NPD group was given normal pellet diet and NAFLD group was given high fat diet, in which 10 % lard (w/w), 10 % yolk powder, 2 % cholesteroland, 0.2 % cholate were added into normal diet [14]. After 4 weeks, all rats were fasted overnight for 12 h, and retro orbital bleeding was conducted for analysis of TG, TC, LDL-C, HDL-C, ALT, and AST in plasma. Besides, five rats in each group were sacrificed randomly. The livers were collected, and pathological changes in the liver tissues were examined by H&E staining.

With successfully making the NAFLD model, the rats in NAFLD group were randomized into four groups of 20 rats each. This four groups were given high-fat diet and normal group (negative control, *n* = 20) was given normal diet as before. they were given rat mash and water ad libitum, and subjected to different dosages of the TFSC viz: group A (positive control) received an equivalent volume of water, group B, 100 mg/Kg body weight of TFSC, group C, 200 mg/Kg body weight of TFSC, group D.400 mg/Kg body weight of TFSC and group E (negative control) received water and normal standard feeds for 6 weeks.

### Blood and tissue sample processing

After the experimental period, the rats were sacrificed following 16 h of starvation, by decapitation. The liver were removed quickly, weighed, and stored at −70 °C until it was analyzed. Blood samples were collected and centrifuged at 3,000 rpm at 4 °C for 15 min, and the supernatants were stored at −70 °C for biochemical analysis.

### Biochemical analysis

The levels of triglyceride (TG), total cholesterol (TC), low-density lipoprotein cholesterol (LDL-C), high-density lipoprotein cholesterol (HDL-C), alanine transaminase (ALT), aspartate aminotransferase (AST), total bile acid (TBA) and blood glucose (GLU) in the serum were measured by the enzymatic colorimetric method using commercial Reagents assay kits (Beijing Biosino Bio-technology & Science Inc, China); The serum concentration of glutathione peroxidase (GSH-Px), Total superoxide dismutase (T-SOD), malondialdehyde (MDA), lactate dehydrogenase (LDH) and Leptin were analyzed using a commercial assay kit (Nanjing Jiancheng Bioengineering Institute, China) according to the manufacturer’s instructions.

### Hepatic lipids analyses

Hepatic lipids were extracted using the method of Bligh and Dyer [15], with slight modifications. Briefly, hepatic tissues (500 mg) were homogenized in 1.5 mL of 0.9 % saline, and 7.5 mL of methanol: chloroform (2:1, v:v) was added to the homogenates. The mixture was shaken horizontally for 10 min and centrifuged at 2,000 × g for 10 min. The lower chloroform phase was withdrawn, and the lipid in this phase was dried and weighed. TC and TG concentrations were determined by enzymatic colorimetric methods using commercial kits as described above.

### Histological analysis of the liver

After six weeks of intervention, the rats were sacrificed and their livers were fixed by 10 % buffered formalin and embedded in paraffin. The liver sections (5 μm) were stained with hematoxylin and eosin (H&E), and the steatoic degrees of individual liver samples were examined and scored, according to the percentage of hepatocytes containing lipid droplets [2, 16] by a pathologist in a blinded manner.

### Extraction and analysis of RNA and analysis of gene expressions

Total RNA was extracted from liver tissue samples using TRIZOL (Invitrogen, USA) according to the manufacturer’s protocol. RNA from Liver samples were extracted for analyzing ACC, CPT-1, PPAR-α, PPAR-γ, SREBP-1c, FAS, LEP and CYP7A1 gene expression, 3.0 μg of the total RNA was reverse-transcribed by Revert Aid First Strand cDNA synthesis kit (TaKaRa Bio Inc.). Real-time quantitative PCR was performed using the SYBRPremix Ex TaqTM (TaKaRa Bio Inc.) according to the manufacturer’s instructions on ABI 7900HT real-time PCRsystem (Applied Biosystems, Foster, CA, USA) with primers as shown in Table 1. The ΔΔCt method was used for relative quantification. The ΔΔCt value for each sample was determined by calculating the difference between the Ct value of the target gene and the Ct value of the β-actin reference gene. The normalized level of expression of the target gene in each sample was calculated using the following formula:

**Table 1:** Primers used for quantitative real-time polymerase chain reaction (PCR)

Gene	Primer pairs	Sequence( 5′ → 3′)	GenBank no	Product length(bp)
CYP7A1	Sense primer	AAAGCGGGAAAGCAAAGACCA	NM_012942	157
	Antisense primer	AGTTCAAAGCAGGAGAGCATCAGG		
CPT-1	Sense primer	CCGCAAACTGGACCGAGAAGAGAT	AF029875	185
	Antisense primer	TTTGCCTGGGATGCGTGTAGTGTT		
PPAR-α	Sense primer	TACCTGTGAACACGATCTGA	NM_013196	136
	Antisense primer	GCTAGTCTTTCCTGCGAGTA		
PPAR-γ	Sense primer	TGTGGGGATAAAGCATCAGC	NM_001145366	175
	Antisense primer	CAAGGCACTTCTGAAACCGA		
SREBP-1c	Sense primer	AGGAGGCCATCTTGTTGCTT	AF286470	126
	Antisense primer	GTTTTGACCCTTAGGGCAGC		
ACC	Sense primer	GGAAGACCTGGTCAAGAAGAAAAT	NM_022193	142
	Antisense primer	CACCAGATCCTTATTATTGT		
β-actin	Sense primer	GGCACCACACTTTCTACAAT	NM_031144	123
	Antisense primer	AGGTCTCAAACATGATCTGG		
Leptin	Sense primer	TGCCTATCCACAAAGTCCAG	NM_013076.2	381
	Antisense primer	TGCTCAGAGCCACCACCT		
FAS	Sense primer	CGGCGTGTGATGGGGCTGGTA	NM_017332	145
	Antisense primer	AGGAGTAGTAGGCGGTGGTGTAGA		

1 $$ {2}^{- \varDelta \varDelta Ct} $$

### Statistical analysis

All values were expressed as mean ± SD and were analyzed using the statistical package for thesocial science (SPSS, version 20). Duncan multiple values test and students t–test were used to detect differences in the mean values of treatment groups and control at significance level of 0.05.

## Results

**Fig. 1:**
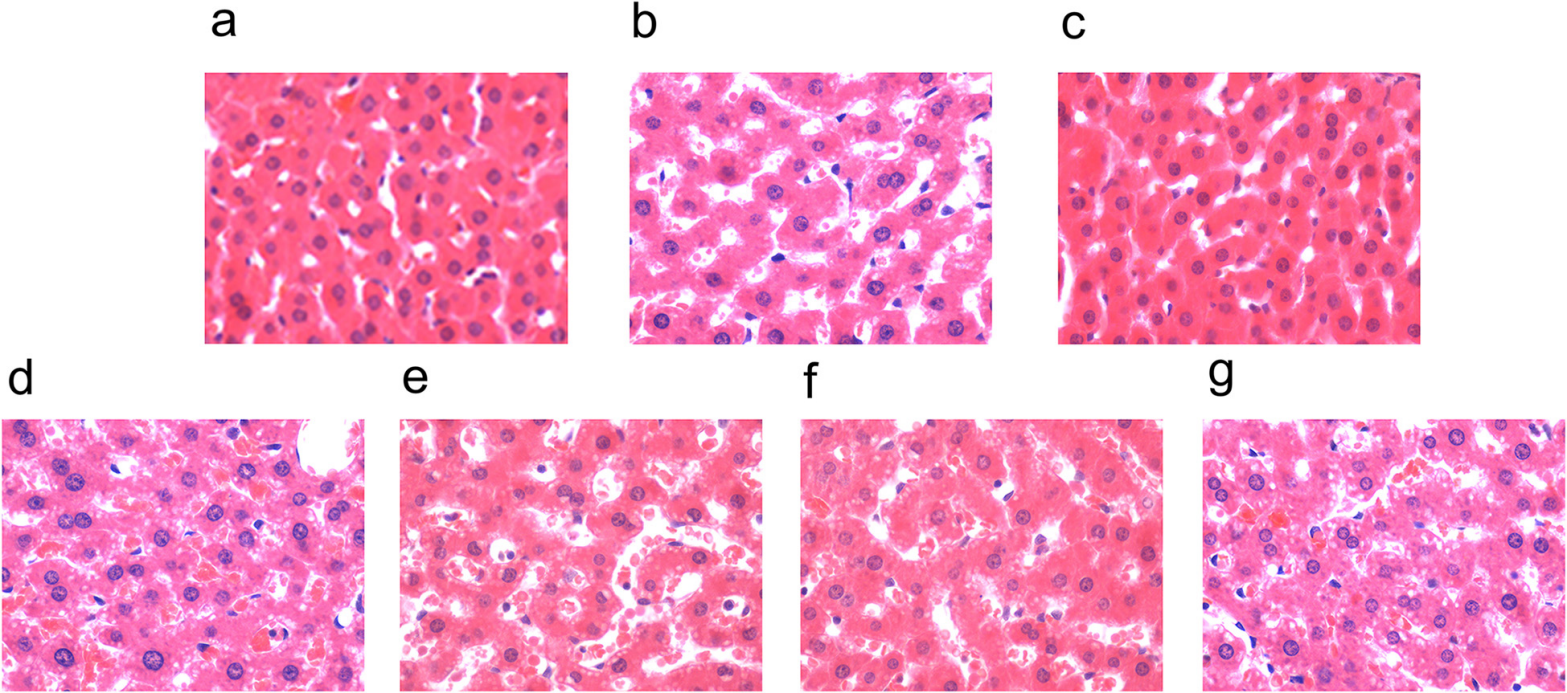
H&E staining for histological evaluation. Typical photographs of liver sections of normal rat (**a**) and NAFLD rat (**b**) in 4 weeks; (**c**)normal rat (negative control group); (**d**) the rat treated with TFSC (100 mg/kg) for 6 weeks; (**e**) the rat treated with TFSC (200 mg/kg) for 6 weeks; (**f**) the rat treated with TFSC (400 mg/kg) for 6 weeks; (**g**) positive control group. (Magnification 400x)

The feeding of high-fat diet for 4 weeks effectively induced NAFLD in rats, as evidenced by the markedly increased body weight, liver weight, and serum TG, TC, LDL-C, ALT, AST and TBA, while decreasing HDL-C (Table 2). Furthermore, H&E staining results (Fig. 1(b)) showed that steatosis was developed and numerous lipid droplets were observed in all of the five livers from the NAFLD rats. This high-fat-diet-induced NAFLD model has the same key pathological features as those reported for other rat NAFLD models [3, 17, 18]. Based on the previous results, the NAFLD model was considered to be successfully established.

**Table 2:** Effect of total flavonoids in *Stellera chamaejasme L.* on weight (g) of rats (mean ± SD) (*n* = 20)

Parameter	4w		6w				
	Negative control group	NAFLD group	Negative control group	Low-dose group	Middle-dose group	High-dose group	Positive control group
Body weight (g)	324.86 ± 24.17	341.45 ± 17.2^★^	365.35 ± 15.23	393.33 ± 23.17^*^	380.61 ± 23.87^*#^	373.52 ± 18.88^#^	396.89 ± 21.22
Liver weight (g)	10.29 ± 0.98	14.39 ± 1.06^★★^	11.65 ± 0.98	18.53 ± 1.26^**^	16.04 ± 1.17^**#^	14.65 ± 1.08^*##^	19.53 ± 1.25
Liver/body weight (%)	3.17 ± 0.17	4.21 ± 0.23^★★^	3.19 ± 0.23	4.71 ± 0.12^**^	4.16 ± 0.22^*#^	3.87 ± 0.19^*##^	4.92 ± 0.35

^★^ *P* < 0.05, ^★★^ *P* < 0.01, compared to normal control group (4w). *P < 0.05, **P < 0.01, compared to normal group (6w); ^#^ *P* < 0.05, ^##^ *P* < 0.01, compared to positive control group (6w)

In the following 6 weeks, rats of high-dose group and middle-dose group showed significantly reduced body weight (by 5.89 % and 4.10 % respectively (*P* < 0.05 for both), liver weight (by 24.99 %, *P* < 0.01 and 17.87 %, *P* < 0.05 respectively), and liver index (liver/body weight, by 21.02 %, *P* < 0.01 and 15.10 %, *P* < 0.05 respectively) compared with rats with the positive control group (NAFLD rats). No statistical difference was found between Low-dose group and the positive control group (Table 2).

Results of AST, ALT, TG, TC, LDL-C, HDL-C, GLU,TBA, Leptin, concentration of rats administered different doses of TFSC are shown in Tables 3. Rats administered 400 mg/kg body weight of TFSC had significant (*P* < 0.01) decrease in TG, TC and MDA compared with the positive control group, while the groups administered 200 mg/kg and 100 mg/kg did not differ significantly in TG and TC, but had decreased and MDA compared with the positive control group (*P* > 0.05). Rats administered 400 mg/kg body weight of TFSC had significant (*P* < 0.05) decrease in AST, ALT, LDL-C and TBA compared with the positive control group, while the groups administered 200 mg/kg and 100 mg/kg did not differ significantly in AST, ALT, LDL-C, but had decreased TBA compared with the positive control group (*P* > 0.05). Rats administered 400 mg/kg body weight of TFSC had significant (*P* < 0.05) increase in HDL-C compared with the positive control group, while the groups administered 200 mg/kg and 100 mg/kg did not differ significantly in HDL-C compared with the positive control group (*P* > 0.05). Meanwhile, hepatic histopathological examination showed the steatosis area and the number of lipid droplets were decreased in the different doses of TFSC-treated group, indicating the improvement of liver steatosis (Fig. 1d, e, f).

**Table 3:** Effect of total flavonoids in *Stellera chamaejasme L.* on serum lipid level in rats (mean ± SD) (*n* = 20)

Parameter	4w		6w				
	Normal group	NAFLD group	Normal group	Low-dose group	Middle-dose group	High-dose group	Positive control group
AST (U/L)	75.17 ± 4.31	96.85 ± 7.63^★★^	76.29 ± 5.16	97.36 ± 6.82**	93.75 ± 4.55*	88.34 ± 6.27*^#^	101.67 ± 8.36
ALT(U/L)	28.16 ± 1.21	51.28 ± 3.26^★★^	29.25 ± 1.72	53.28 ± 4.41**	49.78 ± 2.19**	44.29 ± 2.22**^#^	56.35 ± 4.16
TG(mmol/L)	0.59 ± 0.09	1.65 ± 0.08^★★^	0.61 ± 0.04	1.81 ± 0.11**	1.46 ± 0.09**^#^	0.92 ± 0.03**^##^	2.12 ± 0.12
TC(mmol/L)	1.66 ± 0.12	3.14 ± 0.13^★★^	1.70 ± 0.09	3.82 ± 0.15**	3.65 ± 0.17**^#^	2.65 ± 0.12**^##^	4.54 ± 0.24
LDL-C (mmol/L)	0.54 ± 0.03	1.25 ± 0.08^★★^	0.56 ± 0.04	1.37 ± 0.08**	1.24 ± 0.05**	1.09 ± 0.06**^#^	1.35 ± 0.09
HDL-C (mmol/L)	0.96 ± 0.04	0.64 ± 0.04^★★^	0.98 ± 0.05	0.64 ± 0.03**	0.69 ± 0.05**	0.77 ± 0.04*^#^	0.55 ± 0.03
GLU (mmol/L)	4.68 ± 0.15	5.07 ± 0.21	4.56 ± 0.18	4.99 ± 0.87	5.07 ± 0.04	5.13 ± 18.88	5.41 ± 0.19
TBA(μmol/L)	6.75 ± 0.05	10.23 ± 0.06^★★^	6.54 ± 0.06	11.53 ± 1.27**	11.04 ± 0.98**	10.24 ± 0.05**^#^	12.27 ± 1.25
Leptin (μg/L)	0.18 ± 0.01	0.32 ± 0.01^★^	0.19 ± 0.02	0.46 ± 0.03**	0.34 ± 0.02*^#^	0.28 ± 0.01^#^	0.46 ± 0.03
SOD (U/mL)	374.36 ± 17.86	300.4 ± 20.1^★★^	370.35 ± 19.46	293.4 ± 24.1**	321.79 ± 23.16*	348.32 ± 19.76^#^	297.84 ± 23.17
MDA (nmol/mL)	3.26 ± 0.21	4.27 ± 0.16^★★^	3.25 ± 0.75	4.98 ± 1.32*	4.77 ± 0.87*	3.84 ± 0.99^##^	5.04 ± 1.27
GSH-Px (U/mL)	256.21 ± 19.25	210.45 ± 14.2^★^	255.98 ± 20.54	206.88 ± 15.3**	211.57 ± 17.45**^#^	238.78 ± 14.25^#^	194.21 ± 17.35

^★^ *P* < 0.05, ^★★^ *P* < 0.01, compared to normal control group (4w). *P < 0.05, **P < 0.01, compared to normal group (6w); ^#^ *P* < 0.05, ^##^ *P* < 0.01, compared to positive control group (6w). Normal Group: the group was given normal diet (*n* = 20) for 4 weeks; NAFLD Group: the group was given high-fat diet (*n* = 20) for 4 weeks;Negative Control Group: After the normal diet of 4 weeks, the group was given normal diet (*n* = 20) for 6 weeks; Low-dose group: After the HFD of 4 weeks, the group was administered TFSC orally once a day at a low dose of 100 mg/kg (*n* = 20) for 6 weeks; Middle-dose group: After the HFD of 4 weeks, the group was administered TFSC orally once a day at a medium dose of 200 mg/kg (*n* = 20) for 6 weeks; High-dose group: After the HFD of 4 weeks, the group was administered TFSC orally once a day at a high dose of 400 mg/kg (*n* = 20) for 6 weeks; Positive control group: After the HFD of 4 weeks, the group was given high-fat diet (*n* = 20) for 6 weeks

The results showed that TFSC significantly increased in T-SOD, GSH-Px activity and reduced in MDA level both in rat serum.

**Fig. 2:**
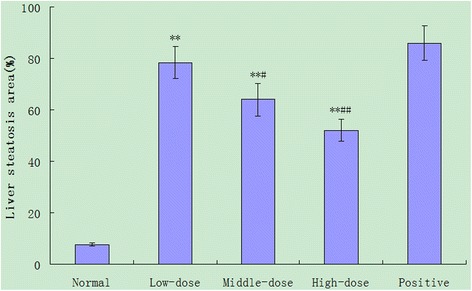
Scores of hepatic steatosis of rat livers. The scores were determined, according to the the percentage of hepatocytes containing lipid droplets. Data are expressed as mean ± SD of each group (*n* = 20 per group) and determined by a pathologist in a blinded fashion. *P < 0.05, **P < 0.01, compared to normal control group, # *P* < 0.05, ## *P* < 0.01, compared to positive control group

After stained with H&E, the degrees of hepatic steatosis were examined. While there was no obvious steatosis in the liver of the normal group of rats, different degrees of liver steatosis were observed in other group rats (Figs. 1 and 2). The area of hepatic steatosis in the high-dose group decreased remarkably when compared with that in the positive control group (*P* < 0.01), in the middle-dose group decreased when compared with that in the positive control group (*P* < 0.05), the score of hepatic steatosis in the low-dose group decreased when compared with that in the positive control group (*P* > 0.05), however, they remained significantly higher than that of the high-dose group or middle-dose group. There was no significant difference in the score of liver steatosis between the high-dose group and middle-dose group (*P* > 0.05) (Figs. 1 and 2).

**Fig. 3:**
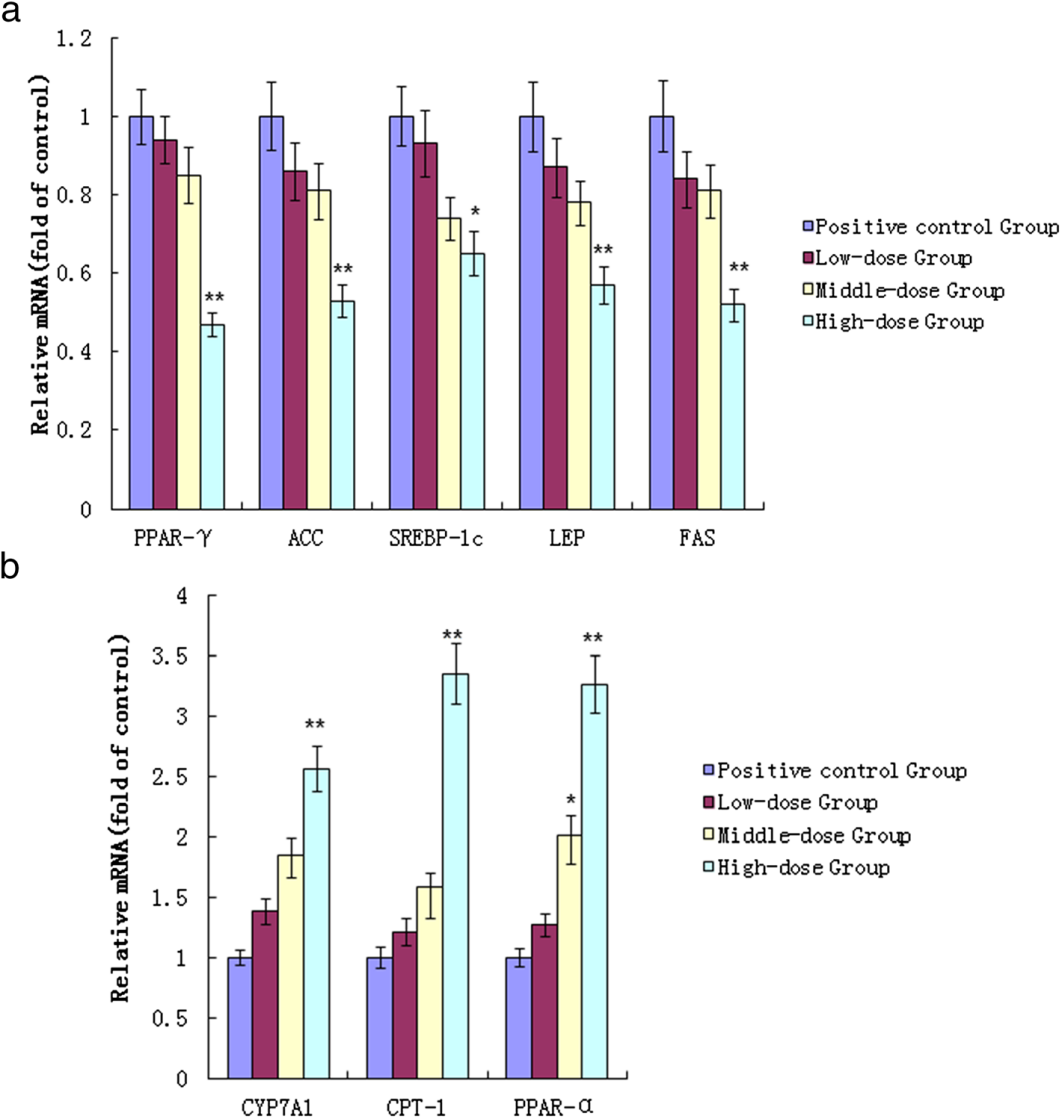
The expression of lipid metabolic regulators in the liver. (**A**) The expression of hepatic PPAR-γand related genes. The relative expression levels of hepatic PPAR-γ, ACC, SREBP-1c, LEP and FAS mRNA transcripts were determinedby real-time PCR in liver samples of rats from the positive control group (NAFLD rats) and the different doses of TFSC-treated group (n =20 per group). (**B**) The expression of CYP7A1and related genes. The relative expression levels of hepatic CYP7A1, CPT-1 and PPAR-α mRNA transcripts were determined by real-time PCR in liver samples of rats from the positive control group (NAFLD rats) and the different doses of TFSC-treated group (n =20 per group). Values are means ± positive and negative errors. Differences between groups were determined by Student’s t test. *P<0.05; **P<0.01 versus the positive control group (NAFLD rats). Abbreviations: CYP7A1, Cholesterol 7α-hydroxylase1; CPT, carnitine palmitoyltransferase; PPAR, peroxisome proliferator-activated receptor; SREBP, sterol regulatory element-binding protein; ACC, Acetyl-CoA carboxylase, FAS, Fatty acid synthase, LEP, Leptin

We next examined whether the different doses of TFSC supplementation affected mRNA expression of genes involved in hepatic lipid metabolism in liver samples of rats. The mRNA levels of genes such as PPAR-γ, SREBP-1c, ACC, FAS and LEP showed a 53 %, 35 %, 47 %, 48 % and 43 % decrease, respectively, in the high-dose group compared with those of the positive control group (NAFLD rats) (Fig. 3a). the mRNA levels of PPAR-γ, SREBP-1c, ACC, FAS and LEP showed a 15 %, 26 %, 19 %, 19 % and 22 % decrease, respectively, in middle-dose group, compared with those of the positive control group (NAFLD rats) (Fig. 3a). Also, the mRNA levels of PPAR-γ, SREBP-1c, ACC, FAS and LEP showed a 6 %, 7 %, 14 %, 16 % and 13 % decrease, respectively, in the low-dose group, compared with those of the positive control group (NAFLD rats) (Fig. 3a). The mRNA levels of PPAR-α, CPT-1, and CYP7A1 showed 3.26-, 3.35-, and 2.56-fold increase, respectively, in the high-dose group compared to the positive control group (Fig. 3b). The mRNA levels of PPAR-α, CPT-1, and CYP7A1 showed 2.01-, 1.58-, and 1.85-fold increase, respectively, in the middle -dose group compared to the positive control group (Fig. 3b). The mRNA levels of PPAR-α, CPT-1, and CYP7A1 showed 1.27-, 1.21-, and 1.38-fold increase, respectively, in the low-dose group compared to the positive control group (Fig. 3b).

Our studies indicated that TFSC had lowering lipid and hepatoprotective effect, and regulated and improved the balancing effects of free radicals on rat model of NAFLD. It’s possible mechanism is that TFSC downregulating the mRNA expression of transcription factor SREBP-lc to regulate the downstream lipid synthesis related genes ACC, FAS and LEP expression, meanwhile, TFSC upregulated the level of transcription factor PPAR-α to regulate the downstream lipidolysis related genes CPT-1 and CYP7A1 expression, and improved the liver tissue β oxidation and in vivo fatty acid degradation, inhibit fatty acid synthesis, and finally reach the effective regulation of lipid metabolisms of experimental rat model of NAFLD (Table 4).

**Table 4:** Effects of Total Flavonoids in *Stellera chamaejasme L.* on liver lipid level in rats (mean ± SD) (*n* = 20)

Group	Hepatic TC (mg/g)	Hepatic TG (mg/g)
Normal group	0.89 ± 0.06	1.52 ± 0.11
Low-dose group	3.86 ± 0.26**	2.56 ± 0.16
Middle-dose group	3.16 ± 0.27**^#^	2.36 ± 0.12
High-dose group	2.19 ± 0.13** ^##^	1.78 ± 0.09^##^
Positive control group	4.37 ± 0.24	2.97 ± 0.19

*P < 0.05, **P < 0.01, compared to normal group (6w); ^#^ *P* < 0.05, ^##^ *P* < 0.01, compared to positive control group (6w)

## Discussion

NAFLD is a common chronic liver disease worldwide and its incidence is increasing in developed countries [3]. Over-consumption of high calories of foods, particularly HFD, is crucial for the development of NAFLD. The treatments of NAFLD currently include oral drug therapy, TCM therapy and exercise therapy. All of these existing traditional drug treatments seem to be inefficiant, therefore it is necessary to find drugs with better effect and fewer side-effects. Recently, many studies have shown that some active ingredients in crude chemicals have stable efficacy in prevention and treatment of NAFLD, and with less toxicity and fewer adverse reactions, which can be made long-term use of (references). Among these, the total flavonoids in *Stellera chamaejasme L.* (TFSC) and its derivatives with its unique physical and properties has been research hotspot of the world since 1980s, and has made big progress in medicine, food and chemical industry, environmental protection, agriculture and many other fields [19, 20]. Recent studies have shown that treatment with flavonoids also benefits patients with NASH and NAFLD [21], However, the mechanisms responsible for lipid-lowering actions of total flavonoids in *Stellera chamaejasme L.* (TFSC) on liver remains unclear. In this study, we explored the efficacy of the different doses of TFSC supplements treatment on the HFD-induced hepatic steatosis and hyperlipidemia in rats. We found that high-dose group or middle-dose group treatment for 6 weeks significantly inhibited the HFD-induced liver weights ,body weights and reduced the ratios of liver to body weights in the NAFLD rats. Furthermore, high-dose group or middle-dose group treatment mitigated the HFD-induced hepatic steatosis, which was associated with the improvement of liver and systemic lipid profiles and function, leading to reduction in the severity of hyperlipidemia in NAFLD rats. Whether TFSC treatment for a longer period could prevent the development of NAFLD-related liver fibrosis and how TFSC treatment could improve lipid metabolism in advanced NAFLD, remain an interest for future investigation.

In this study, high-dose TFSC supplementation in rats fed high fat diet was effective in decreasing liver triglyceride and total cholesterol, and in increasing serum SOD and GSH-Px. This study observed a decline in the mRNA expressions of lipogenic enzymes such as ACC and FAS in high fat with TFSC treatment group. A significant decrease in the transcriptional factor of SREBP-1c, PPAR-γ expression with TFSC supplement was also found in this study. Therefore, lowered hepatic triglyceride and cholesterol levels after TFSC supplementation in high fat fed rats seem to be related with decreased transcriptional factor, SREBP1-c and PPAR-γ which inhibits, in turn, gene expressions of lipogenic enzymes such as ACC and FAS, thus resulting in inhibiting accumulation of triglycerides or cholesterol in liver. Lipid biosynthesis usually occurs in hepatic tissue and typically involves the following lipogenic enzymes: ACC and FAS [22]. In this process, SREBP-1c and PPAR-γ function as an important transcriptional factor that mediates and activates fatty acid and cholesterol biosynthesis pathways [23, 24]. It is believed that SREBP-1c and PPAR-γ greatly increase the expression of genes related with lipogenic pathway in liver [24, 25]. In this study, TFSC supplement led to increase CPT1 expression in the TFSC supplement supplemented group than the high-fat diet group. Since CPT1 mediates the transport of acyl-CoA across the membrane and stimulates the oxidation of long chain fatty acids [25], it suggests that TFSC exhibited hepatic lipid -lowering property which may be also partly mediated via increase of hepatic CPT1 expression.

To understand how hepatic cholesterol content decreased by TFSC treatment, the genetic expression of cholesterol metabolism regulating enzymes, such as CYP7A1, were examined in this study. Human CYP7A1 gene defect can cause cholesterol accumulation in the liver, which has been associated with hypercholesterolemia [26]. This study observed a significant increase in CYP7A1 expression in the TFSC-treated group than the high-fat diet group. This result indicates that TFSC-treated consumption increases CYP7A1 expression in liver and thus reducing cholesterol accumulation in liver.

## Conclusion

In this study, the HFD in the experiment can significantly increase the body weight ,serum TC and TG levels, cause severe lesions and fat mold in rat, showing that the experimental fatty method was feasible and can meet the demand of our research. The TFSC significantly reduced the body weight and the serum biochemical indexes in NAFLD rats, suggesting that it is the effective Chinese medicine on NAFLD rat. The molecular mechanism may be due to the down-ragulation of the level of PPAR-γ mRNA, ACC mRNA, SREBP-1c mRNA, LEP mRNA, FAS mRNA, and the up-ragulation of the level of CYP7A1 mRNA, CPT-1 mRNA and PPAR-α mRNA. The metabolomic results not only provided a systematic view of the development and progression of NAFLD, but also suggested a theoretical basis for the prevention or treatment of NAFLD.

## Abbreviations

CYP7A1: Cholesterol 7α-hydroxylase1
CPT: Carnitine palmitoyl transferase
PPAR: Peroxisome proliferator-activated receptor
SREBP: Sterol regulatory element-binding protein
ACC: Acetyl-CoA carboxylase
FAS: Fatty acid synthase
LEP: Leptin

## References

[1] Angulo P (2002). Nonalcoholic fatty liver disease. N Engl J Med.

[2] Ji G, Zhao X, Leng L, Liu P, Jiang Z (2011). Comparison of dietary control and atorvastatin on high fat diet induced hepatic steatosis and hyperlipidemia in rats. Lipids Health Dis.

[3] Li J, Yang J, Cui W, Chen X, Chen G, Wen X, Wang Q (2013). Analysis of therapeutic effect of ilex hainanensis merr. extract on nonalcoholic fatty liver disease through urine metabolite profiling by ultraperformance Liquid chromatography/ quadrupole time of flight mass spectrometry. Evidence-Based Complementary Alternative Med.

[4] Clark JM (2006). The epidemiology of nonalcoholic fatty liver disease in adults. J Clin Gastroenterol.

[5] Fan JG, Farrell GC (2009). Epidemiology of non-alcoholic fatty liver disease in China. J Hepatol.

[6] Gao Y, Song L, Jiang M, Ge G, Jia Y (2008). Effects of traditional Chinese medicine on endotoxin and its receptors in rats with non-alcoholic steatohepatitis. Inflammation.

[7] Liu W, Wang C (2010). Research on chemical constituents, bioactivity and application of Stellera chamaejasme. Xian Dai Yao Wu Yu Lin Chuang.

[8] Zhao M, Gao X, Wang J, He X, Han B (2013). A review of the most economically important poisonous plants to the livestock industry on temperate grasslands of China. J Appl Toxicol.

[9] Anonymous (1975). Langdu. Quan Guo Zhong Cao Yao Hui Bian.

[10] Liu G, Fu Y, Hou F, Wan L, Wan S, Li M (1995). Chemical constituents of *Stellera chamaejasme L*. Zhong guo Zhong Yao Za Zhi.

[11] Feng B, Pei Y, Hua M (2002). Chemical constituents of *Stellera chamaejasme L*. J Asian Nat Prod Res.

[12] Wang M, Jia ZP, Ma J, Wang B (2005). The antitumor effects of total-flavonoid from *Stellera chamaejasme L*. Zhongguo Zhong Yao Za Zhi.

[13] Wu X, Han M, Liu X, Zhang R, Wang Y (2009). Comparison on the three methods of extracting total flavone from Stellera chamaejasme. Animal Husbandry Feed Sci.

[14] Liu W (2007). Study on blood-fat-lowering effects of phytosterol esters in hyperlipidemia rats.

[15] Bligh EG, Dyer WJ (1959). A rapid method of total lipid extraction and purification. Can J Biochem Physiol.

[16] Janes RG, Prosser M (1947). Influence of high fat diets on alloxan diabetes. Am J Physiol.

[17] Li H, Wang L (2011). A proton nuclear magnetic resonance metabonomics approach for biomarker discovery in nonalcoholic fatty liver disease. J Proteome Res.

[18] Milagro FI, Campión J, Martínez JA (2006). Weight gain induced by high-fat feeding involves increased liver oxidative stress. Obesity (Silver Spring).

[19] Zhang R (2009). Extraction of the active components from Stellera Chamaejrime L. and its effects on immune functions of mice. Inner mongolia agricultural university master's degree thesis.

[20] Chen M (2009). Studies of acarocidal activity and mechanism of the extraction of Stellera Chamaejrime L.

[21] Hu C (2011). Protective effects of total fiavonoids litsea careana an alcoholic fatty liver in rats and the potential mechanism.

[22] Bettzieche A, Brandsch C, Hirche F, Eder K, Stangl GI (2008). L-cysteine down-regulates SREBP-1c-regulated lipogenic enzymes expression via glutathione in HepG2 cells. Ann Nutr Metab.

[23] Foretz M, Guichard C, Ferré P, Foufelle F (1999). Sterol regulatory element binding protein-1c is a major mediator of insulin action on the hepatic expression of glucokinase and lipogenesis-related genes. Proc Natl Acad Sci U S A.

[24] Brown MS, Goldstein JL (1997). The SREBP pathway: regulation of cholesterol metabolism by proteolysis of a membrane-bound transcription factor. Cell.

[25] Ae Wha H, Woo Kyoung K (2013). The effect of fucoxanthin rich power on the lipid metabolism in rats with a high fat diet. Nutr Res Pract.

[26] Woo MN, Jeon SM, Shin YC, Lee MK, Kang MA, Choi MS (2009). Anti-obese property of fucoxanthin is partly mediated by altering lipid-regulating enzymes and uncoupling proteins of visceral adipose tissue in mice. Mol Nutr Food Res.

